# The submerged plus maze as an assay for studying anxiety-like behaviour in fish

**DOI:** 10.1016/j.mex.2019.07.002

**Published:** 2019-07-08

**Authors:** Brittany V. Hope, Trevor J. Hamilton, Peter L. Hurd

**Affiliations:** a Neuroscience and Mental Health Institute, University of Alberta, 4-120 Katz Group Centre, Edmonton, AB T6G 2E1, Canada; b Department of Psychology, Room 6-329, City Centre Campus, MacEwan University, 10700 – 104 Ave., Edmonton, AB T5T 4S2, Canada; c Department of Psychology, University of Alberta, P217 Biological Sciences Building, Edmonton, AB T6G 2E9, Canada

**Keywords:** Submerged plus maze, Elevated plus maze, Anxiety-testing, Behavioural testing, Fish behaviour, Anxiolytic, Anxiogenic

## Abstract

The elevated plus maze is a commonly used and well-validated test of anxiety-related behaviour in rodents. The use of fish in behavioural neuroscience paradigms is increasing, necessitating an equivalent test for studying anxiety-like behaviour in fish. Because behaviour in the elevated plus maze is driven by aversion to open space, the submerged plus maze described here uses transparent walls to elicit similar behaviour in fish. The tendency of fish to explore or avoid the sections of the maze containing transparent walls is used as proxy for anxiety level. This submerged plus maze was designed and validated for convict cichlid (*Amatitlania nigrofasciata*) fish.

## Graphical abstract

**Figure:**
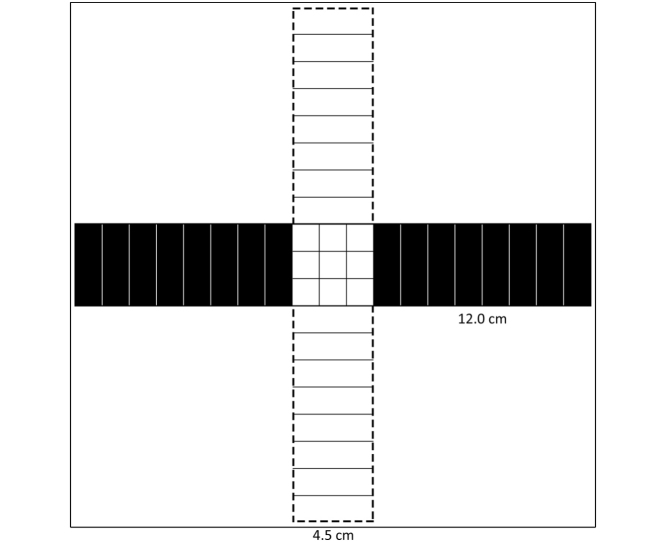

****Value of the Protocol****

**Table:** 

• Fish are increasingly more prevalent in anxiety research. • The submerged plus maze is an aquatic adaptation of the rodent elevated plus maze. • The submerged plus maze can be used to identify anxiolytic or anxiogenic drugs in fish.

**Specifications Table**

**Table:** 

Subject Area:	Neuroscience
More specific subject area:	Animal behaviour
Protocol name:	Submerged Plus Maze
Reagents/tools:	Fish, dip net, apparatus, acclimation chamber, tank water
Experimental design:	Anxiety-like behaviour examined in an aquatic plus maze, where transparent walls elicit behaviours as a result of aversion to open spaces.
Trial registration:	
Ethics:	Protocols were approved by the University of Alberta Biological Sciences Animal Policy and Welfare Committee (protocol number 00000055) and adhere to the guidelines of the Canadian Council for Animal Care.

## Description of protocol

**Fig. 1:**
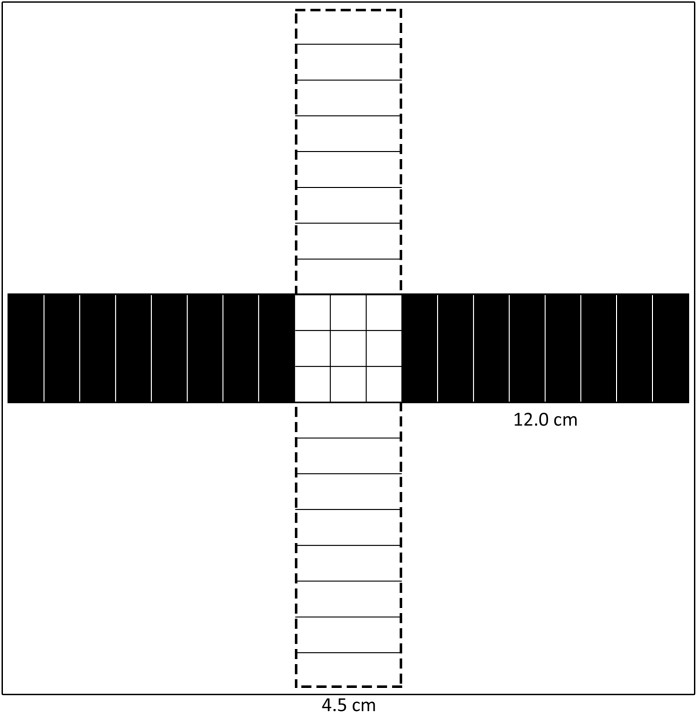
Submerged plus maze apparatus schematic. The apparatus is shaped as a plus symbol with alternating black (black fill) and transparent (white fill, dashed lines) arms. Arms (12 cm long × 4.5 cm wide) are marked to quantify travel within the maze. Fish were placed in an acclimation chamber in the centre area for two minutes before they were released to explore the maze for five minutes.

**Fig. 2:**
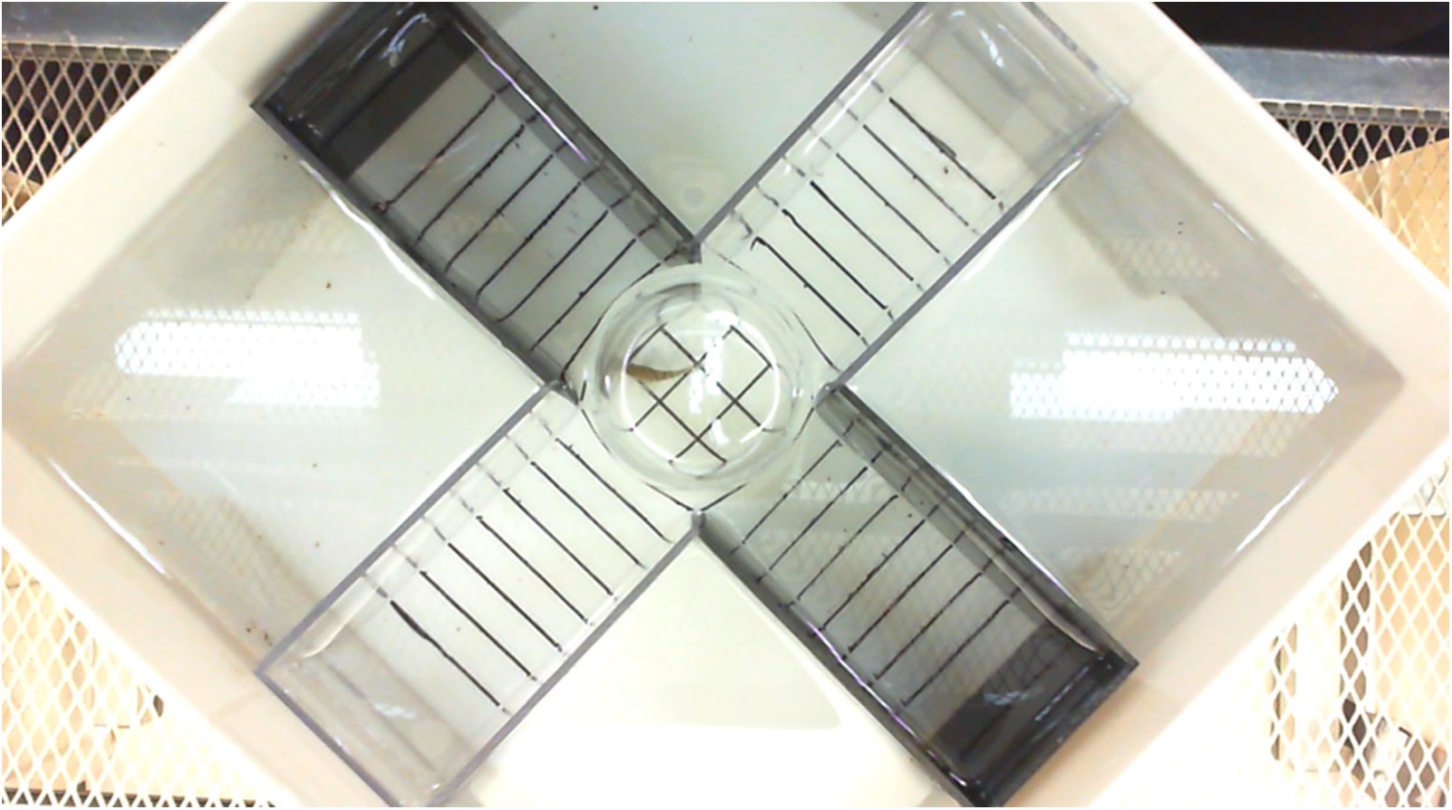
Submerged plus maze apparatus photograph. This photo was taken during the acclimation period, when the fish is placed in a transparent cylinder for two minutes before being released to explore the maze.

The elevated plus maze is used to study anxiety-like behaviour in rodents [1,2] by examining aversion to open spaces (i.e., open arms relative to closed arms) [2]. Studying anxiety using fish is becoming more popular and, as such, an equivalent test should be available. To date, there have been two aquatic mazes using a plus maze structure with four arms [[3], [4], [5]]. Walsh-Monteiro and colleagues [3] constructed a maze with two arms containing ramps that decreased the height of the water column with increasing distance from the center of the maze. In a second maze, Sackerman et al. [4] alternated the arms with white and dark walls, similar to a light/dark preference test [4,5]. Although both of these mazes use a ‘plus’ format, neither are analogous to an elevated plus maze in rodents because of its dependence on aversion to open spaces [2]. Most fish, like rodents, display a preference for dark areas vs. light [6], either for cover [7] or to have a dark background to hide against [8], likely for its potential to provide more protection from predators [9]. The submerged plus maze, as described here, uses aversion to open spaces as an indication of anxiety-like behaviour in fish [3]. This apparatus uses a plus maze format, which includes alternating arms constructed of black or transparent plexiglass walls (each arm 12 cm × 4.5 cm × 13 cm, see Fig. 1, Fig. 2). This arrangement results in two visually closed arms, two visually open arms, and a center area similar to that of the elevated plus maze. The arms are marked in 1.5 cm increments to quantify travel in each area. A transparent plastic cylinder 4.5 cm × 4.5 cm × 13 cm) serves as an acclimation chamber and is placed in the centre area at the start of each trial. A video recording device views the maze from above to record the movements of the fish in the maze for later behavioural scoring. The present study used convict cichlids (*Amatitlania nigrofasciata*) to validate the test, however, the submerged plus maze is also amenable to testing other fish species. Individual fish of the same species may display strong biases for making turns to either the right or left depending on the individual, and these biases can be sensitive to a fish’s shy-vs-bold personality and to the fish’s perceived probability of impending positive or negative outcomes [10,11]. This apparatus has the advantage of being symmetrical, an arm of the opposing type to that currently occupied is immediately to the right and left when exiting the current arm. This should minimize cerebral lateralization biases, but researchers may benefit by recording whether turns are biased to either the right or left when exiting each type of arm.

## Methods

### Behaviour testing

1 Set up plus maze apparatus with a video recorder mounted above the maze so that all areas can be seen clearly. Ensure all areas of the maze are well-lit while reducing glare.
  a Experimenter should be occluded from view.
2 Fill maze with aerated stock tank water or clean water from the fish habitat to a depth of 10 cm and place clear acclimation chamber in the centre area of the maze. Ensure the water matches the physical-chemical properties (temperature, pH, etc.) of habitat water to reduce the risk of climate shock. Specifically, for convict cichlids, we recommend that the water is as follows: pH 7–8, temperature 25 ± 1 °C.
3 Remove fish from home tank using a dip net and deposit in acclimation chamber of the plus maze. Record handling time.
4 Leave fish in the acclimation chamber for two minutes.
  a Start the video recording before the two minutes are fully elapsed (i.e., at 1.5 min).
5 Remove the acclimation chamber, allowing the fish to move freely within the maze. Record behaviour for 5 min.
  a Do not make any loud noises that may disturb the fish.
  b Allow the recording to run slightly past 5 min in case water movement from the removal of the chamber prevents immediate detection of the fish.
6 Return the fish to its home tank.
7 If possible, water should be changed between fish. Otherwise, the temperature, pH, etc. of the water in the arena should be maintained and monitored throughout testing and aerated in between trials. If the temperature drops too low (Δ˜3 °C) or water chemistry changes too drastically, then the arena should be filled with new water or adjusted.
  a The arena should also be rotated after every trial to eliminate any spatial biases or any other uncontrolled auditory or visual stimuli.

### Behaviour scoring

1 Score behaviour using an automated behaviour tracking software (e.g., EthoVision (Noldus), etc.) if possible.
  a This may be difficult if there is too much light glare on the surface of the water or if the fish does not contrast well enough with its background.
2 If the experimenter is scoring behaviour manually, take note of the time the fish enters each arm and how many lines the fish crosses within each arm.
3 Sum the amount of time the fish occupies each area and the number of lines crossed in each of the three areas (i.e., visually closed arms, visually open arms, and centre area).
4 Count the number of entries into new arms and into visually open arms.

### Validation

**Fig. 3:**
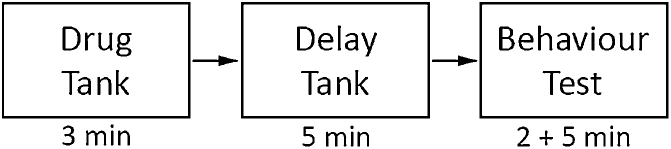
Anxiolytic validation procedure. A fish is first placed into a tank containing the drug treatment (diazepam + vehicle or vehicle only) for 3 min. Next, the fish was moved to a delay tank (tank water) for 5 min to allow the drug to take effect. Finally, the fish was moved to the submerged plus maze test, where it acclimated for 2 min before the 5 min testing period.

This apparatus was validated using the anxiety-reducing (anxiolytic) benzodiazepine diazepam administered by immersion (Fig. 3) [[12], [13], [14], [15]]. Immersion is the method of choice because injection of substances requires anesthesia with MS-222 or cold water, which can impair behavioural responses. Immersion can quickly and reliably be used to validate anxiety-like behavioural tests with other anxiolytic substances (e.g., ethanol) [16], as well as anxiogenic substances (e.g., GABA_A_ receptor antagonist, gabazine) [17,18]. Note that time of immersion in a drug solution can vary from 3 min (e.g., Diazepam) [12,15] up to thirty minutes [16]. If a longer duration of immersion is used, the dosing beaker should be kept under a heating mat to maintain water temperature [16]. To reduce the amount of handling the fish experienced, we used a slotted plastic cup to transport fish between each step. This likely minimizes netting stress and damage to the slime coat of the fish. Fish were housed individually in aquaria partitioned by transparent dividers to control for aggression and social isolation effects and to ensure handling stress was restricted to the fish of interest.

1 Dissolve diazepam (100 mM) in a 0.5% solution of dimethyl sulfoxide (DMSO) in aerated tank water. Then, dilute this solution with aerated tank water to a final volume of 500 mL while maintaining the desired concentration (2.5 mg/L was used in the current study, see Table 1) in the drug administration beaker. Agitate slightly to resuspend the solution.
  a Note: 2.5 mg/L was the concentration used in the current study, determined after an initial pilot test to obtain a dose-response curve. Different sizes and species of fish may respond optimally to different drug concentrations and therefore any new studies should obtain an appropriate dose-response curve.

**Table 1:** Drug solutions for validation. Amount of reagents required for administration through immersion at typical drug concentrations. Vehicle for diazepam was 5% DMSO in tank water and diazepam is soluble in 100 mM DMSO. Dilute diazepam + vehicle solution to a final volume of 500 mL for administration.

Concentration (mg/L)	Diazepam (mg)	DMSO (μL)	Tank Water (mL)	Final Volume (mL)
2.5	1.25	44	8.8	→500
5	2.5	88	17.6	→500
10	5.0	176	35.1	→500
2 Prepare an additional holding container with aerated tank water.
3 Remove the fish from its home tank with a dip net and deposit fish into the slotted plastic cup.
4 Expose the fish to the drug by placing the cup holding the fish in the drug administration beaker for 3 min.
  a Note: the drug solution remains in this beaker and is not transferred to any subsequent steps.
5 Lift the cup and fish and move them into the holding container for 5 min to allow the drug to take effect.
6 Lift the cup and fish and transfer fish into the acclimation chamber of the submerged plus maze by pouring it out of the cup and begin the test.

This validation method was used in a within-subjects design, so fish were exposed to the submerged plus maze with and without prior diazepam administration. In vehicle trials, fish underwent the same steps but with only DMSO and tank water in the drug administration beaker. Drug and vehicle trial order were randomized to ensure behavioural differences between trials was not due to testing order, and we imposed a 48-h inter-trial interval to ensure drug elimination between trials. Note that we used a within-subjects design because of low numbers of fish and high individual variability, however, a between-subjects design can also be used to validate this test with other fish species.

## Additional information

The dimensions of this apparatus were designed to accommodate fish ranging between 1.0–4.5 cm in standard length. An alternate apparatus for fish ranging between 3.0–7.0 cm in standard length was constructed where each arm measured 24 cm × 9 cm × 16.5  cm and lines were marked in 3 cm increments. The maze dimensions should be adjusted accordingly to match target fish size.

A basin should be used to surround the maze and water should flow between the basin and the maze to alleviate pressure against the walls of the plus maze. The present maze employed small gaps at the far end of each arm to exchange water with the surrounding basin. A previous iteration of the maze used gaps too large for some of the smallest fish in our experiments and gauze had to be used to prevent the fish from wedging themselves in the gaps. Smaller gaps were used in a subsequent version, eliminating the issue.

## References

[1] Pellow S., Chopin P., File S.E., Briley M. (1985). Validation of open: closed arm entries in an elevated plus-maze as a measure of anxiety in the rat. J. Neurosci. Methods.

[2] Treit D., Menard J., Royan C. (1993). Anxiogenic stimuli in the elevated plus-maze. Pharmacol. Biochem. Behav..

[3] Walsh-Monteiro A., Pessoa R.D.S., Sanches É.M., Carvalho A.C.C.D., Chirinéa G., Gouveia A. (2016). A new anxiety test for zebrafish: plus maze with ramp. Psychol. Neurosci..

[4] Gould G.G. (2011). Aquatic light/dark plus maze novel environment for assessing anxious versus exploratory behavior in zebrafish (Danio rerio) and other small teleost fish. Neuromethods.

[5] Sackerman J., Donegan J.J., Cunningham C.S., Nguyen N.N., Lawless K., Long A., Benno R.H., Gould G.G. (2010). Zebrafish behavior in novel environments: effects of acute exposure to anxiolytic compounds and choice of Danio rerio line. Int. J. Comp. Psychol./ISCP.

[6] Maximino C., De Brito T.M., de Mattos Dias C.A.G., Gouveia A., Morato S. (2010). Scototaxis as anxiety-like behavior in fish. Nat. Protoc..

[7] Kistler C., Hegglin D., Würbel H., König B. (2011). Preference for structured environment in zebrafish (Danio rerio) and checker barbs (*Puntius oligolepis*). Appl. Anim. Behav. Sci..

[8] Bano F., Gupta P., Agarwal P., Serajuddin M. (2018). Black/White preference and novel tank test to evaluate anxiety in adult goldfish, *Carassius auratus*. Trends Biosci..

[9] Hamilton I.M., Dill L.M. (2002). Monopolization of food by zebrafish (*Danio rerio*) increases in risky habitats. Can. J. Zool..

[10] Reddon A.R., Hurd P.L. (2009). Individual differences in cerebral lateralization are associated with shy–bold variation in the convict cichlid. Anim. Behav..

[11] Reddon A.R., Hurd P.L. (2009). Sex differences in the cerebral lateralization of a cichlid fish when detouring to view emotionally conditioned stimuli. Behav. Processes.

[12] Hope B.V., Hamilton T.J., Hurd P.L. (2019). Submerged plus maze: a novel test for studying anxiety-like behaviour in fish. Behav. Brain Res..

[13] Schnörr S.J., Steenbergen P.J., Richardson M.K., Champagne D.L. (2012). Measuring thigmotaxis in larval zebrafish. Behav. Brain Res..

[14] Gebauer D.L., Pagnussat N., Piato A.L., Schaefer I.C., Bonan C.D., Lara D.R. (2011). Effects of anxiolytics in zebrafish: similarities and differences between benzodiazepines, buspirone and ethanol. Pharmacol. Biochem. Behav..

[15] Bencan Z., Sledge D., Levin E.D. (2009). Buspirone, chlordiazepoxide and diazepam effects in a zebrafish model of anxiety. Pharmacol. Biochem. Behav..

[16] Hamilton T.J., Morrill A., Lucas K., Gallup J., Harris M., Healey M., Pitman T., Schalomon M., Digweed S., Tresguerres M. (2017). Establishing zebrafish as a model to study the anxiolytic effects of scopolamine. Sci. Rep..

[17] Ou M., Hamilton T.J., Eom J., Lyall E.M., Gallup J., Jiang A., Lee J., Close D.A., Yun S.S., Brauner C.J. (2015). Responses of pink salmon to CO_2_-induced aquatic acidification. Nat. Clim. Change.

[18] Hamilton T.J., Holcombe A., Tresguerres M. (2014). CO2-induced ocean acidification increases anxiety in rockfish via alteration of GABAA receptor functioning. Proc. R. Soc. B.

